# Dietary Myo‐Inositol Improves Growth Performance and Glycolipid Metabolism in Yellow River Carp (*Cyprinus carpio haematopterus*) Fed High‐Carbohydrate‐High‐Fat Diets: Involvement of PI3K/Akt and FXR/SHP Pathways

**DOI:** 10.1155/anu/4324808

**Published:** 2026-10-25

**Authors:** Xiao Yan, Xin Long, Rumeng Qiao, Peng Pang, Junli Wang, Shaoyang Zhi, Jiali Mi, Mingming Niu, Chaobin Qin, Liping Yang, Guoxing Nie

**Affiliations:** ^1^ Aquatic Animal Nutrition and Feed Research Team College of Fisheries Henan Normal University Xinxiang 453007 Henan Province China henannu.edu.cn; ^2^ College of Life Sciences Henan Normal University Xinxiang 453007 Henan Province China henannu.edu.cn; ^3^ College of Fisheries Xinyang Agriculture and Forestry University Xinyang 464000 Henan Province China xyafu.edu.cn

**Keywords:** FXR/SHP pathway, glycolipid metabolism, high-carbohydrate-high-fat diet, myo-inositol, PI3K/Akt pathway, Yellow River carp

## Abstract

High‐carbohydrate‐high‐fat (HCHF) diets often induce glycolipid metabolism disorders, Hepatosomatic fat deposition, and compromised growth in fish. Myo‐inositol (MI) is a known metabolic regulator, yet its specific mechanism in mitigating HCHF‐induced metabolic stress in Yellow River carp remains unclear. This study investigated the effects of dietary MI on growth, glycolipid metabolism, and bile acid signaling in fish fed HCHF diets. Six experimental diets were formulated: a control diet, an HCHF diet, and the HCHF diet supplemented with graded levels of MI (125, 250, 500, and 1000 mg·kg^−1^). After a 9‐week feeding trial, results showed that MI supplementation dose‐dependently improved growth performance (FBW, WGR, and SGR) and feed utilization (FCR), while significantly reducing the hepatosomatic index (HSI). Physiologically, MI reduced serum glucose and triglycerides (TGs), as well as hepatopancreatic total cholesterol (T‐CHO) and TG levels, while elevating high‐density lipoprotein cholesterol (HDL‐C), suggesting enhanced lipid transport capacity. Regarding glycolipid metabolism, MI promoted glycolysis by increasing *hk* and *pfk* mRNA expression, while suppressing gluconeogenesis (*pepck*) and lipogenesis (*fas*, *pparγ*). These metabolic changes were accompanied by alleviated hepatocyte vacuolation and morphological abnormalities, with reduced hepatocellular vacuolation and lipid deposition in HCHF‐fed fish. Mechanistically, bile acid metabolism and phosphatidylinositol 3‐kinase (PI3K)/Akt (protein kinase B) signaling pathways participated extensively and served important roles in the action of MI. Based on comprehensive analysis of growth and physiological indicators, the optimal MI supplementation level in the HCHF diet is recommended as 230–475 mg·kg^−1^. In conclusion, MI alleviates HCHF diet‐induced metabolic disorders and hepatopancreatic morphological abnormalities in Yellow River carp, and this effect is associated with the PI3K/Akt and farnesoid X receptor (FXR)/SHP (small heterodimer partner) pathways, providing a theoretical basis for functional feed development for fish.

## 1. Introduction

With improvements in living standards, the global demand for aquatic products has increased, and aquaculture has made a significant contribution to global fish production and consumption [1]. Moreover, capture fisheries production has not increased in the past two decades, and aquaculture production must expand to meet the growing demand for fisheries products from a larger and more affluent human population. Since about two‐thirds of farmed finfish and crustaceans rely on formulated feeds, the expansion of aquaculture has, in turn, driven the rapid development of the aquaculture feed industry [2]. However, to pursue high production, intensive aquaculture relies heavily on high‐energy‐formulated feeds. These practices lead to frequent fish diseases and excessive fat accumulation, resulting in mass fish mortality and severely hindering the resilient and sustainable development of aquaculture systems [3, 4]. In view of this, in recent years, the exploration and development of green and natural feed additives to mitigate the adverse effects of aquaculture have become a research hotspot in this field.

Inositol, also referred to as cyclohexanehexol, is a water‐soluble cyclic‐saturated hexahydroxycyclohexane and an essential nutrient for animals, plants, and microorganisms. Inositol exists in nine stereoisomeric forms (myo‐, scyllo‐, muco‐, neo‐, D‐chiro‐, L‐chiro‐, allo‐, cis‐, and epi‐inositol). Among them, myo‐inositol (MI) is the most prevalent and biologically significant isomer in animals [5]. MI, as a precursor of phosphatidylinositol and lipids, has key biological functions in somatic growth regulation, membrane lipid synthesis, and cellular signal transduction [6]. As a second messenger in various intracellular signal transduction pathways, MI modulates multiple signaling cascades associated with glycolipid metabolism [7], while it can enhance the antioxidant capacity of organisms. Studies conducted by Katayama [8] demonstrated that MI could significantly inhibit the increase in liver weight of rats induced by a high‐glucose diet and effectively alleviate hepatic lipid accumulation caused by nonalcoholic fatty liver disease. In fish‐related studies, dietary supplementation with 1200 mg·kg^−1^ MI in a high‐glucose diet was found to markedly increase the hepatic glucose metabolism capacity in tilapia. Moreover, it promoted glucose utilization by stimulating insulin secretion, thereby mitigating lipid accumulation induced by high‐glucose feed and improving the osmoregulatory ability of tilapia [9]. Supplementation with MI significantly reduced fat deposition in the whole body, hepatopancreas, and muscle of juvenile *Eriocheir sinensis* and alleviated high‐fat diet‐induced lipid accumulation. Moreover, the expression levels of key genes involved in lipid synthesis in the hepatopancreas (e.g., *fas* and *acc*) were significantly downregulated, which inhibited de novo lipogenesis and intestinal lipid absorption [10]. Collectively, these findings provide a solid theoretical basis for the application of MI in the regulation of glycolipid metabolism in fish.

In recent years, the application of high‐carbohydrate‐high‐fat (HCHF) diets in the artificial culture of Yellow River carp has posed enormous challenges to aquaculture that prioritizes fish health, safe aquatic products, and sustainable production. Previous studies have demonstrated that MI can improve the stress resistance and immune function of carp [11]. However, energy metabolism is closely associated with fish health. However, systematic research and detailed reports on the effects of MI on glycolipid metabolism in Yellow River carp are currently lacking. This deficiency not only hinders the in‐depth elucidation of the potential regulatory functions of MI in common carp physiology but also restricts its optimized application in practical aquaculture to a certain extent.

Therefore, this study was conducted using Yellow River carp as the animal model. By supplementing the HCHF diet with MI at different concentrations, we systematically analyzed the effects of MI on growth performance, hepatopancreas histomorphology, activities of enzymes related to glycolipid metabolism, the bile acid metabolic pathway, and the expression levels of key genes and proteins involved in glycolipid metabolism in Yellow River carp. The objectives of this study were to reveal the potential molecular mechanisms underlying the MI‐mediated regulation of glycolipid metabolism in Yellow River carp and to determine the optimal MI supplementation level in HCHF diets. This work is expected to provide a scientific basis for the rational application of MI in the healthy culture of Yellow River carp while laying a theoretical foundation for improving the utilization efficiency of carbohydrates and lipids in fish feeds, reducing feed costs, and enhancing aquaculture benefits.

## 2. Materials and Methods

### 2.1. Animal Ethics Statement

All experimental procedures involving animals adhered to ethical guidelines and were approved by the Institutional Animal Care and Use Committee of Henan Normal University (Approval No. HNSD‐2026BS‐0303).

### 2.2. Diet Preparation and Feeding Trial Design

Six diets were used in this study: a control diet (CON), a HCHF diet, and four HCHF diets with graded MI supplementation at 125, 250, 500, and 1000 mg·kg^−1^ (Solarbio, China). The corresponding groups were abbreviated as MI 125, MI 250, MI 500, and MI 1000. The ingredient formulations and nutritional levels of each group are presented in Table 1.

**Table 1:** The composition and nutrient content of the experimental diets.

Component (%)	CON	HCHF	MI 125	MI 250	MI 500	MI 1000	
Fish meal	10	10	10	10	10	10	
Meat meal	5	5	5	5	5	5	
Soybean meal	20	20	20	20	20	20	
Corn gluten meal	18.5	18.5	18.5	18.5	18.5	18.5	
Wheat meal	7	7	7	7	7	7	
Corn starch	15	29	29	29	29	29	
Soybean oil	2.6	8.2	8.2	8.2	8.2	8.2	
Monocalcium phosphate	0.8	0.8	0.8	0.8	0.8	0.8	
Vitamin premix^a^	0.07	0.07	0.07	0.07	0.07	0.07	
Mineral premix^b^	0.11	0.11	0.11	0.11	0.11	0.11	
Choline chloride	0.2	0.2	0.2	0.2	0.2	0.2	
Carboxy methyl cellulose	0.8	0.8	0.8	0.8	0.8	0.8	
Cellulose microcrystalline	19.72	0.12	0.1075	0.095	0.07	0.02	
Lysine	0.2	0.2	0.2	0.2	0.2	0.2	
MI	0	0	0.0125	0.025	0.05	0.1	
Proximate composition, measured value (%)							
Moisture	9.35	8.80	9.47	8.99	9.07	9.35	
Crude protein	31.95	32.5	32.6	32.34	32.38	32.34	
Crude lipid	4.37	10.01	10.53	10.47	10.88	10.11	
Crude ash	7.05	6.98	7.44	7.69	7.77	7.71	
MI	0.015	0.014	0.025	0.038	0.062	0.112	

^a^Vitamin premix (per kg of premix): VA 2,000,000–6,000,000 IU, VD3 1,000,000–2,000,000 IU, VE 9000–30,000 mg, VK3 2700–9000 mg, VB1 550–1800 mg, VB2 860–2880 mg, VB6 5500–18,000 mg, VB12 10–20 mg, nicotinamide 10,000–42,000 mg, folic acid 900–3000 mg, calcium pantothenate 5500–18,000 mg, biotin 20–60 mg, VC 6000–20,000 mg, and inositol 7200–24,000 mg.

^b^Mineral premix (per kg of premix): Cu 3000–12,000 mg, Fe 5000–20,000 mg, Zn 8000–32,000 mg, Mn 10,000–40,000 mg, I 200–800 mg, and Se 100–400 mg.

The control diet in this experiment was formulated according to the Chinese national standard GB/T 22919.28 (Aquafeed—Part 28: Formula feed for common carp) and the published diet formulas for Yellow River carp [12]. It matches the physiological and nutritional requirements for normal growth of this species and stably supplies nutrients required for normal physiological metabolism, serving as the baseline representing the normal physiological status in this study. The HCHF diet was prepared by increasing dietary fat and carbohydrate levels to create nutritional stress and establish a metabolic disorder model for exploring physiological and molecular responses induced by the HCHF diet.

During diet preparation, all dry ingredients were ground and then sieved through a 60‐mesh screen. Subsequently, oil and water were added according to the formulated proportions and mixed thoroughly. Finally, the mixture was processed into pellets with a diameter of 1.5 mm using a feed pellet mill. The dietary MI levels in this trial were determined according to the optimal supplementation dosages reported in previous studies on aquatic animals [13–15].

### 2.3. Experimental Fish Management

The experimental fish were reared in pond cages located in Luohe city, Henan Province, China. A total of 540 experimental fish with an initial body weight of 56.58 ± 0.41 g were randomly allocated into 18 cages (2 m × 2 m × 1.5 m), with 30 fish per cage, corresponding to a stocking density of 5 fish m^−3^. Before the formal feeding trial, the fish were acclimated for 2 weeks and fed three times daily at 7:30, 12:00, and 18:00. During the acclimation period, the dissolved oxygen (DO) concentration in the pond was maintained above 5 mg·L^−1^, and the pH was maintained within the range of 7.0–7.5. Water quality parameters were monitored daily throughout the feeding trial, and aerators were turned on and off as needed to sustain adequate DO levels. After successful acclimation, the formal feeding trial was initiated, which lasted for 63 days. The fish were fed daily at a feeding rate of 3% of their initial body weight. The feed ration was calculated based on the individual initial body weight measured at the start of the trial. No further capture and weighing were performed during the rearing period. Instead, the feed amount was updated weekly according to the estimated weight gain of experimental fish, based on our previous experience with Yellow River carp reared in net cages. This strategy avoids stress induced by frequent capture and weighing and maintains an appropriate feeding level.

### 2.4. Sample Collection

At the end of the feeding trial, all experimental fish were anesthetized with MS‐222 at a concentration of 55 mg·L^−1^. Fish in each cage were counted, and their body weight, visceral weight, and hepatopancreas weight were measured to calculate growth performance parameters.

Blood samples were collected from the caudal vein of fish in each group and then centrifuged at 3500 × *g* for 10 min at 4°C, and the serum was aspirated and stored at −80°C for later use. Hepatopancreas, intestinal, and muscle tissue samples were rapidly dissected under sterile conditions, immediately frozen in liquid nitrogen, and then stored at −80°C. Hepatopancreas samples were used for the detection of gene and protein expression, glycogen content, and enzyme activities. Intestinal samples were used for the determination of gene expression and lactic acid (LA) content. Muscle samples were used for glycogen content analysis. Hepatopancreas tissue samples were collected from fish in each group and fixed in 4% paraformaldehyde for histomorphological analysis.

### 2.5. Growth Performance

Growth performance and feed utilization efficiency were calculated according to the following formulas:

Survival rate=Final number of surviving fish⋅initial number of fish−1×100,

Weight gain rate WGR,%=Wt - W0⋅W0−1×100,

Specific growth rate SGR,%⋅day−1=100×lnWt -  lnW0⋅t−1,

Feed conversion ratio FCR=F⋅Wt - W0−1,

Viscerosomatic index VSI,%=Visceral weight⋅Wt−1×100,

Hepatosomatic Index HSI,%=Hepatopancreas weight⋅Wt−1×100,

Condition factor CF,%=Wt⋅Lt−3×100,

where *W*_0_ is the initial body weight of the fish at the start of the trial (g); *W*_*t*_ is the final body weight of the fish at the end of the trial (g); *F* is the dry weight of total feed intake (g); *L*_*t*_ is the final body length of the fish at the end of the trial (cm); and *t* is the experimental duration (day).

### 2.6. Histomorphological Analysis

After being fixed in 4% paraformaldehyde, the hepatopancreas tissue samples were embedded in paraffin and sectioned into 4 μm‐thick slices, followed by hematoxylin–eosin (H&E) staining and periodic acid–Schiff (PAS) staining. Random visual fields per sample were selected for observation and photography under a light microscope.

### 2.7. Enzyme Activities and Biochemical Assays

Kits for the detection of glucose (Glu, Cat. No. F006‐1‐1), triglyceride (TG, Cat. No. A110‐2‐1), total cholesterol (T‐CHO, Cat. No. A111‐1‐1), high‐density lipoprotein cholesterol (HDL‐C, Cat. No. A112‐2‐1), low‐density lipoprotein cholesterol (LDL‐C, Cat. No. A113‐1‐1), LA (Cat. No. A019‐2‐1), glutamic pyruvic transaminase (GPT, Cat. No. C009‐1‐1), glutamic oxaloacetic transaminase (GOT, Cat. No. C010‐1‐1), total superoxide dismutase (T‐SOD, Cat. No. A001‐1‐2) and glycogen (Cat. No. A043‐1‐1) were detected using biochemical assay kits (Njjcbio, China). Kits (Solarbio, China) for the determination of the enzyme activities of hexokinase (HK, Cat. No. BC0745), pyruvate kinase (PK, Cat. No. BC0540), phosphoenolpyruvate carboxykinase (PEPCK, Cat. No. BC3310) and phosphofructokinase (PFK, Cat. No. BC0530), as well as H&E staining solution (Cat. No. G1120), PAS glycogen staining solution (Cat. No. G1281) and BCA protein assay kit (Cat. No. PC0020) were used to determine the physiological and biochemical parameters and enzyme activities in the serum, hepatopancreas, and muscle tissues.

For the determination of tissue enzyme activities, 0.1 g of the tissue sample was weighed and placed in a 1.5 mL sterile enzyme‐free centrifuge tube. Physiological saline was added at a ratio of 1:9 (tissue mass, g AND saline volume, mL). After homogenization, the mixture was centrifuged at 3500 rpm for 10 min at 4°C. The supernatant was collected, aliquoted into 200 μL sterile enzyme‐free tubes, and stored at −80°C for subsequent use. The protein concentrations of the tissue samples were determined using a BCA protein assay kit. The enzyme activities of the tissue samples were calculated on the basis of the measured optical density (OD) values and protein concentrations.

### 2.8. Total RNA Extraction and Quantitative Real‐Time PCR (RT‐qPCR)

Total RNA was extracted from tissue samples using RNAiso Plus (RNA extraction reagent, Cat. No. 9109, Takara, Japan). The concentration and purity of the RNA samples were determined with a Nanodrop 2000 spectrophotometer, with the OD_260_/OD_280_ ratio required to be within the range of 1.8–2.0 to ensure RNA quality. The complementary DNA (cDNA) used was the PrimeScript RT reagent Kit with the gDNA Eraser (Cat. No. RR092A, Takara, Japan).

RT‐qPCR assays were performed using the SupRealQ Purple Universal SYBR qPCR Master Mix (U+) (Cat. No. Q412−02, Vazyme, China) on a Light Cycler 480 instrument (Roche, Switzerland). The primer sequences are listed in Table S1. The 18S rRNA gene was used as the internal reference gene, and data analysis was conducted using the 2^−ΔΔCt^ method [16].

### 2.9. Western Blotting

Proteins were extracted from hepatopancreas tissues using RIPA lysis buffer supplemented with protease and phosphatase inhibitors. The protein concentration was determined using a BCA protein assay kit (Cat. No. A043–1–1, Solarbio, China). After denaturation by boiling, the proteins were separated using sodium dodecyl sulfate–polyacrylamide gel electrophoresis (SDS–PAGE) and then transferred onto 0.45 μm polyvinylidene fluoride (PVDF) membranes. The PVDF membranes were blocked in TBST buffer containing 5% nonfat milk powder for 1 h, followed by incubation with primary antibodies at room temperature for 2 h and subsequent overnight incubation at 4°C. Thereafter, the membranes were incubated with horseradish peroxidase (HRP)‐conjugated secondary antibodies at room temperature for 2 h, and the protein bands were visualized using an enhanced chemiluminescence (ECL) reagent. The intensities of the target protein bands were quantified using ImageJ software. Western blot assays were performed to detect the protein expression levels of farnesoid X receptor (FXR; 1:1000, Cat. No. bs‐12867R, Bioss, China), small heterodimer partner (SHP; 1:1000, Cat. No. bs‐4311R, Bioss, China), CYP7A1 (1:1000, Cat. No. bs‐21430R, Bioss, China), GLUT1 (1:1000, Cat. No. T55360, Abmart, China), PPARγ (1:1000, Cat. No. bs‐4590R, Bioss, China), p‐phosphatidylinositol 3‐kinase (PI3K; 1:1000, Cat. No. AF3242, Affinity Biosciences, USA), p‐Akt (1:1000, Cat. No. AF0016, Affinity Biosciences, USA), PI3K (1:1000, Cat. No. AF6242, Affinity Biosciences, USA), and Akt (1:1000, Cat. No. GB15689, Affinity Biosciences, USA). β‐Actin (1:20,000, Cat. No. 81,115–1‐RR, Proteintech, China) was used as the internal reference antibody.

### 2.10. Regression Curve Analysis

In this study, dietary inositol inclusion level was taken as the independent variable, while growth performance, serum and hepatopancreas physiological–biochemical indices, and key enzyme activities were set as dependent variables. Cubic polynomial regression analysis was performed using GraphPad Prism 9 (USA). The optimal dietary inositol level for each indicator was calculated according to the extreme values of the regression curves, and the suitable inositol inclusion range was predicted by integrating multiple indicators.

### 2.11. Statistical Analysis

Prior to the statistical comparisons, the normality of the data distribution and homoscedasticity were first verified. For comparisons between the CON group and the HCHF group, an independent‐samples *t* test was performed. Differences were considered statistically significant at *p*  < 0.05, which is marked with “ ^∗^”; specifically,  ^∗^ *p*  < 0.05,  ^∗∗^ *p*  < 0.01, and  ^∗∗∗^ *p*  < 0.001. For comparisons between the HCHF group and each MI‐supplemented group, one‐way analysis of variance (one‐way ANOVA) followed by Duncan’s multiple range test (DMRT) was conducted, and significant differences (*p* < 0.05) are indicated with lowercase letters. All the data were expressed as the means ± standard errors of the means (SEM), and the statistical analyses were performed using SPSS 20.0 software (IBM, Chicago, USA).

## 3. Results

### 3.1. Effects of Dietary MI Supplementation on the Growth Performance of Yellow River Carp

The survival rate of all experimental fish reached 100% at the end of the trial. The effects of MI supplementation on the growth performance of Yellow River carp are presented in Table 2. Compared with those of the CON group, the FBW and WGR of the HCHF group were significantly greater (*p* < 0.05). After MI supplementation in the HCHF diet, the FBW, WGR, and SGR of the MI 125 and MI 250 group were significantly greater than those of the HCHF group (*p* < 0.05). Compared with those in the HCHF group, the hepatosomatic index (HSI) was significantly lower in MI 250, MI 500, and MI 1000 group (*p* < 0.05). No significant difference in the CF was observed among the groups (*p* > 0.05).

**Table 2:** Effects of MI on the growth performance of Yellow River carp.

Index	CON	HCHF	MI 125	MI 250	MI 500	MI 1000
IBW (g)	56.58 ± 0.37	56.58 ± 0.55	56.54 ± 0.46	56.57 ± 0.40	56.56 ± 0.32	56.63 ± 0.37
FBW (g)	185.37 ± 2.84	193.79 ± 0.98 ^∗^^b^	203.91 ± 1.31^a^	206.38 ± 2.05^a^	200.05 ± 3.26^ab^	200.80 ± 5.08^ab^
WGR (%)	227.62 ± 5.02	242.5 ± 1.73 ^∗^^b^	260.40 ± 2.32^a^	264.76 ± 3.63^a^	253.58 ± 5.76^ab^	254.89 ± 8.97^ab^
SGR (%·day^−1^)	1.86 ± 0.02	1.92 ± 0.01^b^	2.00 ± 0.01^a^	2.02 ± 0.01^a^	1.97 ± 0.02^ab^	1.98 ± 0.04^ab^
FCR	1.52 ± 0.03	1.43 ± 0.01^a^	1.33 ± 0.01^b^	1.31 ± 0.02^b^	1.36 ± 0.03^ab^	1.36 ± 0.05^ab^
VSI (%)	9.58 ± 0.28	9.51 ± 0.21^ab^	9.79 ± 0.30^ab^	9.92 ± 0.39^ab^	10.55 ± 0.36^a^	9.16 ± 0.38^b^
HSI (%)	2.06 ± 0.09	2.36 ± 0.09^a^	2.32 ± 0.12^a^	2.07 ± 0.07^b^	1.87 ± 0.03^b^	1.9 ± 0.06^b^
CF (g·cm^−3^)	2.54 ± 0.05	2.57 ± 0.03	2.62 ± 0.04	2.58 ± 0.05	2.55 ± 0.04	2.62 ± 0.03

*Note*: Data are expressed as mean values ± SEM. (*n* = 3). Asterisks represent significant differences between the CON and HCHF groups ( ^∗^ *p* < 0.05). Different superscript lowercase letters indicate significant differences between the HCHF group and each MI group (*p* < 0.05).

### 3.2. Effects of MI Supplementation in the HCHF Diet on the Physiological and Biochemical Indices of Yellow River Carp

The physiological and biochemical indices of the serum, hepatopancreas, and gut of Yellow River carp are presented in Table 3.

**Table 3:** Effects of MI on physiological and biochemical indices in the serum, hepatopancreas, and gut of Yellow River carp.

Index	CON	HCHF	MI 125	MI 250	MI 500	MI 1000
Serum						
Glu (mmol·L^−1^)	5.53 ± 0.07	6.61 ± 0.92 ^∗^^a^	6.33 ± 1.08^abc^	5.59 ± 0.68^c^	5.74 ± 0.94^bc^	6.48 ± 1.08^ab^
T‐CHO (mmol·L^−1^)	5.48 ± 0.48	5.86 ± 0.21^ab^	6.03 ± 0.22^ab^	5.74 ± 0.34^ab^	5.54 ± 0.35^b^	6.47 ± 0.28^a^
TG (mmol·L^−1^)	2.02 ± 0.12	2.24 ± 0.14^ab^	2.48 ± 0.16^a^	2.35 ± 0.11^a^	1.9 ± 0.12^b^	2.18 ± 0.15^ab^
LDL‐C (mmol·L^−1^)	1.57 ± 0.17	1.79 ± 0.16	1.66 ± 0.19	1.43 ± 0.10	1.46 ± 0.21	1.32 ± 0.13
HDL‐C (mmol·L^−1^)	2.47 ± 0.15	1.99 ± 0.08^b^	2.32 ± 0.15^ab^	2.41 ± 0.13^a^	2.37 ± 0.17^ab^	2.07 ± 0.08^ab^
GOT (U·L^−1^)	18.17 ± 1.52	24.99 ± 1.69 ^∗^^a^	18.66 ± 1.51^b^	15.88 ± 1.67^bc^	12.72 ± 0.63^c^	13.69 ± 0.97^c^
GPT (U·L^−1^)	17.25 ± 0.29	16.76 ± 0.46	16.94 ± 0.44	17.03 ± 0.21	15.87 ± 0.88	16.57 ± 0.20
T‐SOD (U·mL^−1^)	4.08 ± 0.23	2.20 ± 0.19 ^∗^^b^	2.60 ± 0.25^ab^	3.15 ± 0.13^a^	2.62 ± 0.20^ab^	2.31 ± 0.17^b^
Hepatopancreas						
T‐CHO (μmol·g prot ^−1^)	30.52 ± 4.17	44.79 ± 3.62 ^∗^^a^	44.40 ± 2.62^a^	34.35 ± 4.41^b^	32.09 ± 2.22^b^	33.50 ± 3.26^b^
TG (mmol·g prot^−1^)	0.12 ± 0.01	0.21 ± 0.01 ^∗^^a^	0.18 ± 0.02^ab^	0.15 ± 0.01^b^	0.15 ± 0.01^b^	0.21 ± 0.01^a^
LDL‐C (μmol·g prot^−1^)	6.60 ± 0.47	9.41 ± 3.07 ^∗^	10.21 ± 2.00	8.81 ± 2.30	10.71 ± 5.93	8.60 ± 1.71
HDL‐C (μmol·g prot^−1^)	24.79 ± 2.66	15.77 ± 1.47 ^∗^^b^	17.33 ± 2.09^ab^	15.37 ± 2.30^b^	18.49 ± 1.61^ab^	23.68 ± 3.97^a^
LA (mmol·g prot^−1^)	0.27 ± 0.02	0.31 ± 0.03	0.31 ± 0.01	0.33 ± 0.03	0.30 ± 0.02	0.30 ± 0.01
GOT (U·g prot^−1^)	8.03 ± 1.08	11.54 ± 1.01 ^∗^^a^	10.31 ± 0.75^ab^	9.04 ± 1.52^ab^	8.33 ± 1.20^ab^	8.06 ± 0.76^b^
GPT (U·g prot^−1^)	2.89 ± 0.66	4.81 ± 0.47 ^∗^^a^	3.04 ± 0.63^b^	4.07 ± 0.37^ab^	2.98 ± 0.62^b^	2.94 ± 0.67^b^
T‐SOD (U·mg prot^−1^)	28.72 ± 0.80	25.91 ± 0.69 ^∗^^b^	30.34 ± 1.74^a^	26.69 ± 1.40^b^	25.86 ± 0.64^b^	19.89 ± 1.08^c^
Gut						
LA (mmol·g prot^−1^)	0.14 ± 0.01	0.18 ± 0.01^ab^	0.15 ± 0.01^c^	0.16 ± 0.01^bc^	0.18 ± 0.01^ab^	0.19 ± 0.01^a^

*Note*: Data are expressed as the mean values ± SEM (*n* = 3). Asterisks represent significant differences between the CON and HCHF groups ( ^∗^ *p* < 0.05;  ^∗∗^ *p* < 0.01). Different superscript lowercase letters indicate significant differences between the HCHF group and each MI group (*p* < 0.05).

In serum, compared with CON, HCHF significantly increased Glu level (*p* < 0.05). When compared with HCHF, different doses of MI produced varying regulatory effects. The MI 250 group had significantly lower Glu and higher serum HDL‐C (*p* < 0.05). The levels of T‐CHO and TG were the lowest in the MI 500 group, with no significant difference compared with the HCHF group (*p* > 0.05).

In hepatopancreas, HCHF increased T‐CHO, TG, LDL‐C, GOT, and GPT, and decreased HDL‐C (*p* < 0.05). MI downregulated hepatopancreatic T‐CHO and TG in MI 250 and MI 500 groups, and upregulated HDL‐C in MI 1000 (*p* < 0.05). It also effectively lowered hepatopancreatic GOT and GPT activities.

The HCHF diet significantly decreased T‐SOD activity in serum and hepatopancreas (*p* < 0.05). MI 250 supplementation markedly restored serum T‐SOD activity (*p* < 0.05), while MI 125 significantly elevated hepatopancreatic T‐SOD activity (*p* < 0.05). Notably, T‐SOD activity in the MI 1000 group was significantly lower than that in the HCHF group (*p* < 0.05). Compared with those in the HCHF group, the intestinal LA level in the MI 125 group was significantly lower (*p* < 0.05).

### 3.3. Effects of MI Supplementation in the HCHF Diet on the Gene and Protein Expression of the Bile Acid Metabolic Pathway and Glycolipid Metabolism in Yellow River Carp

The FXR/SHP pathway is a key regulatory pathway for bile acid metabolism as well as glycolipid metabolism. As shown in Figure 1A, after Yellow River carp were fed the HCHF diet, the expression of related genes in the hepatopancreas changed as follows: compared with those in the CON group, the mRNA expression levels of *fxr* and *shp* in the hepatopancreas of the HCHF group were significantly increased (*p* < 0.05), whereas the mRNA expression levels of these two genes in the MI 500 group were significantly lower than those in the HCHF group (*p* < 0.05). Moreover, compared with the CON group, the mRNA expression level of *cyp7a1* and *cyp8b1*, downstream bile acid synthesis‐related gene, in the hepatopancreas of the HCHF group was significantly downregulated (*p* < 0.05), whereas the mRNA expression of *cyp7a1* in the MI 500 group and *cyp8b1* in the MI 250 group was significantly higher than that in the HCHF group (*p* < 0.05).

**Figure 1:**
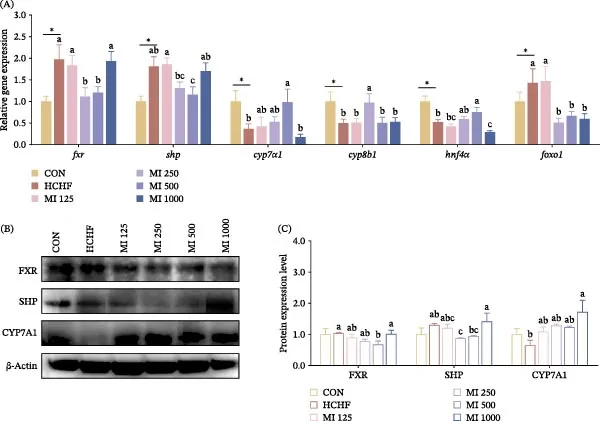
Effects of MI on the expression of bile acid metabolism‐related genes and proteins in the hepatopancreas of Yellow River carp. (A) Expression of bile acid metabolism‐related genes. (B) Expression of bile acid metabolism‐related proteins. (C) Quantitative analysis of bile acid metabolism‐related protein expression. The data are expressed as the means ± SEM (*n* = 3). Asterisks represent significant differences between the CON and HCHF groups ( ^∗^ *p* < 0.05). Different lowercase letters indicate significant differences between the HCHF group and each MI group (*p* < 0.05).

Further analysis of the expression of downstream signaling genes in the FXR/SHP pathway revealed that after the fish were fed the HCHF diet, the mRNA expression level of *hnf4*α in the hepatopancreas was significantly downregulated (*p* < 0.05), whereas that in the MI 500 group was significantly upregulated (*p* < 0.05). In contrast, the mRNA expression level of *foxo1* was significantly increased in the HCHF group (*p* < 0.05), and the mRNA expression levels of *foxo1* in the MI 250, MI 500 and MI 1000 groups were significantly lower than those in the HCHF group (*p* < 0.05). Based on the above gene expression results, western blot analysis was performed on the hepatopancreas tissues. As shown in Figure 1B,C, compared with that in the HCHF group, the protein expression level of FXR was significantly lower in the MI 500 group, whereas that of SHP was significantly lower in the MI 250 group (*p* < 0.05). The SHP protein expression level in the MI 500 group tended to decrease (*p* = 0.056).

The expression of intestinal bile acid metabolism‐related genes in Yellow River carp is shown in Figure 2. The mRNA expression level of *fxr* in the HCHF group was significantly greater than that in the CON group (*p* < 0.05). Compared with those in the HCHF group, the mRNA expression levels of *fxr* in the MI 250, MI 500, and MI 1000 groups were significantly lower, whereas the mRNA expression levels of *tgr5* and *fgf19* in the MI 250 group were significantly greater (*p* < 0.05).

**Figure 2:**
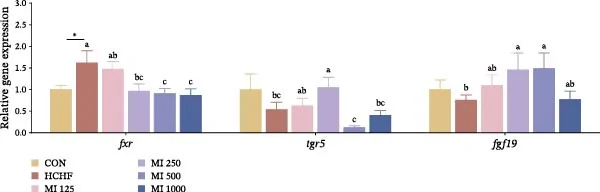
Effects of MI on the expression of bile acid metabolism‐related genes in the intestine of Yellow River carp. The data are expressed as the means ± SEM (*n* = 3). Asterisks represent significant differences between the CON and HCHF groups ( ^∗^ *p* < 0.05). Different lowercase letters indicate significant differences between the HCHF group and each MI group (*p* < 0.05).

Appropriate MI supplementation contributes to the promotion of glycolysis as well as the inhibition of gluconeogenesis. The results of glycolysis‐related key enzyme‐encoding gene expression are shown in Figure 3A. Compared with that in the CON group, the mRNA expression level of the key glycolysis enzyme *hk* in the HCHF group was significantly lower. Compared with those in the HCHF group, the mRNA expression levels of *hk* and *pfk* in the MI 250 group were significantly greater (*p* < 0.05). The results of gluconeogenesis‐related key enzyme‐encoding gene expression are shown in Figure 3A. Compared with those in the CON group, the mRNA expression levels of *fbpase* and *pepck* in the HCHF group were significantly greater (*p* < 0.05). Compared with the HCHF group, MI supplementation significantly downregulated the mRNA expression levels of *fbpase*, *pepck*, and *g6pase* (*p* < 0.05).

**Figure 3:**
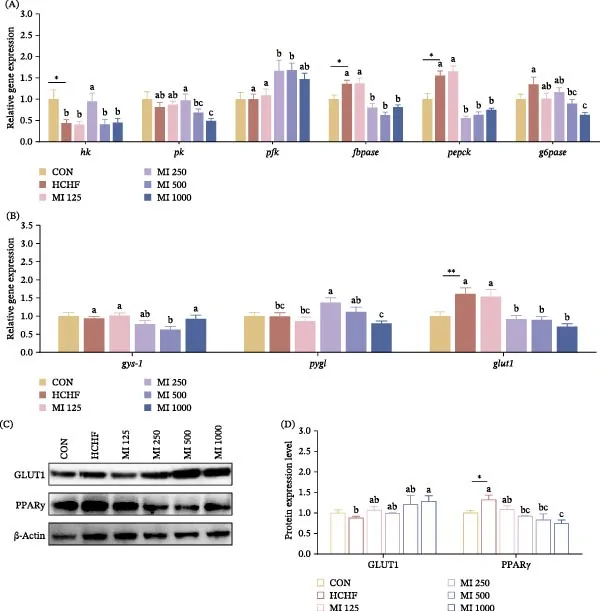
Effects of MI on the expression of glucose metabolism‐related genes and proteins in the hepatopancreas of Yellow River carp. (A and B) Expression of glucose metabolism‐related genes. (C) Expression of glucolipid metabolism‐related proteins. (D) Quantitative analysis of glucolipid metabolism‐related protein expression. The data are expressed as the mean values ± SEM (*n* = 3). Asterisks represent significant differences between the CON and HCHF groups ( ^∗^ *p* < 0.05). Different lowercase letters indicate significant differences between the HCHF group and each MI group (*p* < 0.05).

MI mediates biological processes such as glycogen metabolism and glucose uptake and transport. As shown in Figure 3B–D, the results of this study demonstrated that, compared with the HCHF group, MI upregulated the mRNA expression level of *pygl* but significantly downregulated the mRNA expression levels of *gys-1* and *glut1* (*p* < 0.05), with the most prominent regulatory effect observed in the MI 500 group. Compared with the CON group, PPARγ protein expression was significantly upregulated in the HCHF group (*p* < 0.05), while it was significantly downregulated in the MI 250, MI 500, and MI 1000 groups relative to the HCHF group (*p* < 0.05). Compared with the HCHF group, the expression level of GLUT1 protein was significantly increased in the MI 1000 groups (*p* < 0.05).

Inositol plays an important role in mediating the regulation of glucose metabolism by insulin. As shown in Figure 4A,B, compared with those in the CON group, the mRNA expression levels of *irs-1*, *pi3k*, and *ampka2* were significantly lower, whereas the mRNA expression level of *gcgb* was significantly greater in the HCHF group (*p* < 0.05). After MI supplementation, the mRNA expression levels of *gcga*, *gcgb*, and *gsk3*β were significantly decreased, whereas the mRNA expression levels of *ins*, *irs-1*, *akt*, and *ampka2* were significantly increased (*p* < 0.05). Compared with the HCHF group, there were no significance in the expression levels of *ir* and *ampk1* genes (*p* > 0.05). The protein expression results are shown in Figure 4C,D; compared with those in the HCHF group, MI treatment increased the phosphorylation levels of PI3K and Akt. The above regulatory effects of inositol on glycogen synthesis, insulin metabolism, and energy metabolism were most pronounced in the MI 250 and MI 500 groups.

**Figure 4:**
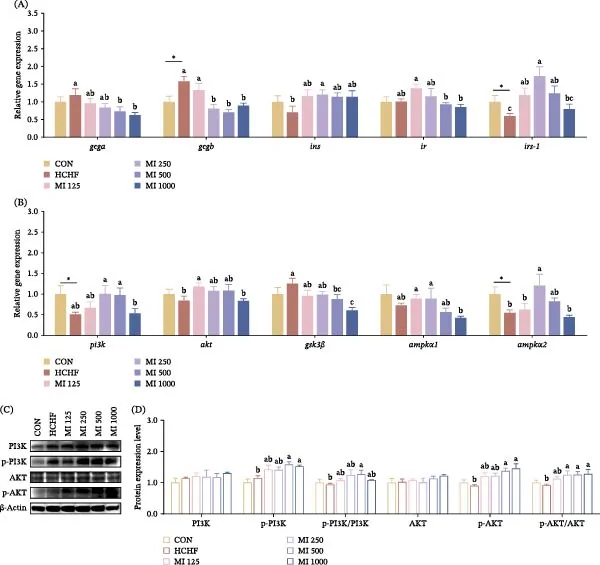
Effects of MI on the PI3K/Akt signaling pathway and the expression of energy metabolism‐related proteins in the hepatopancreas of Yellow River carp. (A, B) Expression of insulin signaling pathway metabolism‐related genes. (C) Expression of insulin signaling pathway metabolism‐related proteins. (D) Quantitative analysis of insulin signaling pathway metabolism‐related protein expression. The data are expressed as the mean values ± SEM (*n* = 3). Asterisks represent significant differences between the CON and HCHF groups ( ^∗^ *p* < 0.05,  ^∗∗^ *p* < 0.01). Different lowercase letters indicate significant differences between the HCHF group and each MI group (*p* < 0.05).

In terms of lipid metabolism regulation, MI can reduce body fat accumulation by inhibiting fatty acid synthesis, promoting fatty acid catabolism, and downregulating the expression of fatty acid transport‐related genes. The specific results are presented below (Figure 5A,B). MI inhibited the expression of fatty acid synthesis‐related genes, including *fas* and *acc-1*, and reduced *srebp-1* expression notably at the MI 250 dose (*p* < 0.05) (Figure 5A). Alongside this, MI promoted lipid metabolism regulation by suppressing the expression levels of lipid storage‐related genes *pparγ* at MI 500 and MI 1000 dose (*p* < 0.05) (Figure 5A). Compared with those in the CON group, the mRNA expression levels of *cd36* and *fatp* in the HCHF group were significantly upregulated, whereas those of *cpt1* were significantly downregulated (*p* < 0.05) (Figure 5B). In contrast, compared with those in the HCHF group, the mRNA expression levels of *cd36* and *fatp* in the MI 250 group were significantly downregulated, whereas those of *hsl*, *cpt1* and *aco* were significantly upregulated (*p* < 0.05) (Figure 5B).

**Figure 5:**
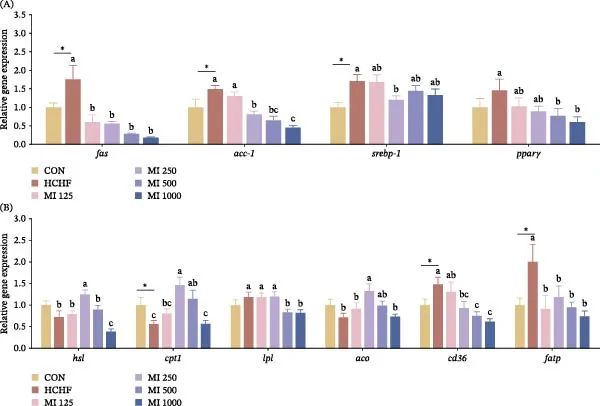
Effects of MI on the expression of lipid metabolism‐related genes in the hepatopancreas of Yellow River carp. (A) Expression of lipogenesis metabolism‐related genes. (B) Expression of lipolysis and lipid transport metabolism‐related proteins. The data are expressed as the means ± SEM (*n* = 3). Asterisks represent significant differences between the CON and HCHF groups ( ^∗^ *p* < 0.05). Different lowercase letters indicate significant differences between the HCHF group and each MI group (*p* < 0.05).

### 3.4. Effects of MI Supplementation in the HCHF Diet on the Activities of Key Glucose Metabolism Enzymes and Glycogen Content in Yellow River Carp

The results of key enzyme activity assays of glucose metabolism revealed that among the glycolytic enzymes, HK activity in the HCHF group was significantly lower than that in the CON group (*p* < 0.05), and MI supplementation had no significant effect on HK activity. The PK activity in the MI 500 group was significantly greater than that in the HCHF group (*p* < 0.05). The PFK activity was the highest in the MI 250 and MI 500 groups, but there was no significant difference compared with that in the HCHF group (*p* > 0.05). Compared with that in the CON group, the PEPCK activity in the HCHF group was significantly greater (*p* < 0.05). In contrast, compared with that in the HCHF group, PEPCK activity in the MI 250 group was significantly lower (*p* < 0.05) (Table 4).

**Table 4:** Effects of MI on glucose metabolism in the hepatopancreas of Yellow River carp.

Index	CON	HCHF	MI 125	MI 250	MI 500	MI 1000
HK (U·mg prot^−1^)	3.83 ± 0.75	1.88 ± 0.39 ^∗^	1.82 ± 0.55	1.84 ± 0.75	2.13 ± 0.88	2.57 ± 0.73
PK (U·mg prot^−1^)	4.30 ± 0.43	3.82 ± 0.4^b^	5.7 ± 0.74^ab^	5.77 ± 0.84^ab^	7.29 ± 0.82^a^	5.01 ± 0.5^b^
PFK (U·mg prot^−1^)	5.84 ± 1.55	3.57 ± 0.64^ab^	2.17 ± 0.76^b^	5.68 ± 1.12^a^	4.73 ± 0.89^a^	3.61 ± 0.55^ab^
PEPCK (U·mg prot^−1^)	77.08 ± 4.97	103.26 ± 3.12 ^∗^^a^	88.05 ± 4.3^ab^	75.11 ± 6.73^b^	97.8 ± 8.08^a^	91.67 ± 8.69^ab^

*Note*: Mean values ± SEM (*n* = 3). Asterisks indicate significant differences between the CON and HCHF groups ( ^∗^ *p* < 0.05). Different superscript lowercase letters indicate significant differences between the HCHF group and each MI group ( ^∗^ *p* < 0.05).

The results of glycogen content detection are presented in Table 5. After Yellow River carp were fed the HCHF diet, their hepatic glycogen content was significantly higher than that in the CON group. Compared with HCHF group, MI supplementation significantly decreased the hepatic glycogen level (*p* < 0.05). No significant difference in muscle glycogen levels was observed among the groups (*p* > 0.05). As shown by the PAS staining results of hepatopancreas glycogen sections (Figure 6), the glycogen content in the HCHF group was significantly higher than that in the CON group, whereas that in the MI‐supplemented groups was significantly lower than that in the HCHF group (*p* < 0.05).

**Figure 6:**
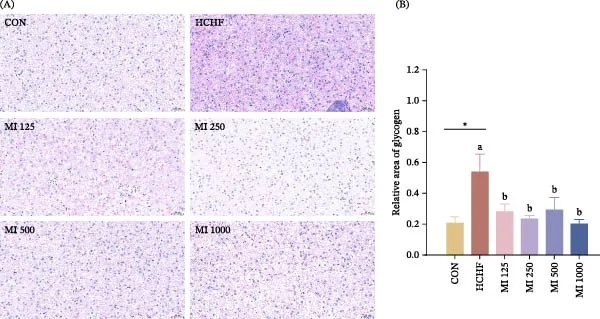
Effects of MI on PAS staining of glycogen in the hepatopancreas of Yellow River carp (10×). (A) PAS staining of the hepatopancreas. (B) Quantitative analysis of the glycogen area. The data are expressed as the mean values ± SEM (*n* = 3). Asterisks represent significant differences between the CON and HCHF groups ( ^∗^ *p* < 0.05). Different lowercase letters indicate significant differences between the HCHF group and each MI group (*p* < 0.05).

**Table 5:** Effects of MI on the hepatopancreas and muscle glycogen contents of Yellow River carp.

Index	CON	HCHF	MI 125	MI 250	MI 500	MI 1000
Hepatopancreas glycogen (mg·g^−1^)	12.43 ± 1.04	15.27 ± 0.70 ^∗^^a^	13.66 ± 0.88^ab^	12.22 ± 0.64^b^	11.28 ± 0.71^b^	12.13 ± 1.06^b^
Muscle glycogen (mg·g^−1^)	0.608 ± 0.04	0.773 ± 0.12	0.575 ± 0.05	0.563 ± 0.05	0.573 ± 0.06	0.613 ± 0.06

*Note*: Mean values ± SEM (*n* = 3). Asterisks indicate that significant differences exist between the CON and HCHF groups ( ^∗^ *p* < 0.05). Different superscript lowercase letters indicate significant differences between the HCHF group and each MI group ( ^∗^ *p* < 0.05).

### 3.5. Effects of MI Supplementation in the HCHF Diet on Histological Morphology of the Hepatopancreas in Yellow River Carp

The results of H&E staining of hepatopancreas tissue sections are shown in Figure 7. Compared with those in the CON group, the hepatocytes in the HCHF group were irregularly arranged with blurred boundaries and exhibited hypertrophy with indistinct nuclear membranes, cytoplasmic vacuolation, and decreased nuclear counts. In contrast, dietary MI supplementation alleviated hepatopancreatic vacuolation. Hepatocytes displayed large, round nuclei with clear and intact nuclear membranes (Figure 7A). The ratio of cytoplasmic vacuolization area to total area was quantified to evaluate hepatocyte swelling, vacuolation, and morphological alterations. The percentage of cell area relative to the total area was significantly higher in the HCHF group compared with the CON group (*p* < 0.05). With increasing dietary MI supplementation, the cell‐area ratio first decreased and then increased. The MI 250 group exhibited the lowest cell‐area ratio, which was significantly lower than that of the HCHF group (Figure 7B).

**Figure 7:**
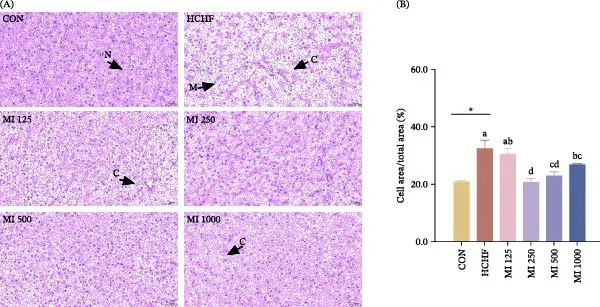
Effects of MI on the histological morphology of the hepatopancreas in Yellow River carp (H&E staining, 40×). (A) H&E staining of the hepatopancreas (N: nucleus, M: cell membrane, C: cytoplasmic vacuolization). (B) Quantitative analysis of the cytoplasmic vacuolization droplet area. The data are expressed as the mean values ± SEM (*n* = 3). Asterisks represent significant differences between the CON and HCHF groups ( ^∗^ *p* < 0.05). Different lowercase letters indicate significant differences between the HCHF group and each MI group (*p* < 0.05).

### 3.6. Regression Analysis

Data on growth performance, serum biochemical indices, and activities of key enzymes related to glycolipid metabolism in the hepatopancreas that exhibited significant intergroup differences were further subjected to cubic polynomial regression analysis. Indicators with a coefficient of determination *R* ^2^ >0.85 were selected to determine the optimal response relationships between these key parameters and dietary MI levels. On the basis of growth performance, the optimal supplementation range of MI in the HCHF diet was determined to be 251–260 mg·kg^−1^ (Figure 8). On the basis of serum and hepatopancreatic enzyme activities and physiological and biochemical indices, the optimal MI supplementation range for the HCHF diet was 230–475 mg·kg^−1^ (Figure 9).

**Figure 8:**
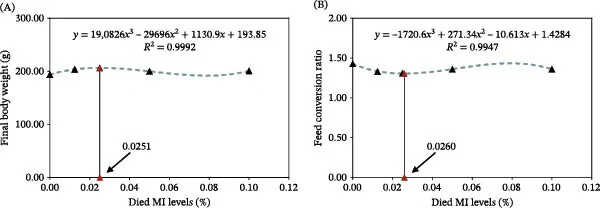
Cubic polynomial regression analysis of growth performance indices, (A) final body weight and (B) feed conversion ratio, against dietary MI levels.

**Figure 9:**
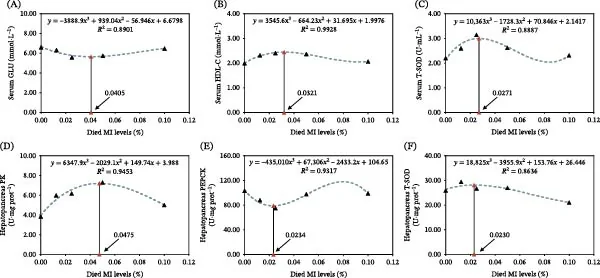
Cubic polynomial regression analysis between hepatopancreatic enzyme activities, serum physiological and biochemical indices and dietary MI levels in Yellow River carp. (A) Blood glucose, (B) serum HDL‐C, (C) serum T‐SOD, (D) hepatopancreatic PK, (E) hepatopancreatic PEPCK, and (F) hepatopancreatic T‐SOD.

## 4. Discussion

In fish, MI is an essential substance for maintaining physiological homeostasis. It participates in a wide range of biological processes, including growth regulation, biomembrane formation, fertilization, hormone secretion, neurotransmitter signal transduction, oxidative stress regulation, and the improvement of muscle quality [17–19]. However, most fish species exhibit limited endogenous capacity for MI synthesis, which cannot fulfil their physiological and metabolic requirements. Accordingly, dietary supplementation with MI is necessary to sustain normal growth and development [20]. In practical aquaculture production, targeted supplementation of inositol in compound feed is highly practical for improving the fish growth rate, maintaining metabolic homeostasis of the organism, and ensuring their normal development [15].

### 4.1. Effects of MI on Promoting the Growth Performance of Yellow River Carp

Current research on MI in aquatic animals has focused mainly on basal diets, with the core objective of determining the optimal MI requirement thresholds for different fish species. However, reports concerning appropriate MI supplementation levels and their related efficacy in practical diets with high sugar and lipid contents, which are more aligned with actual aquaculture production conditions, are scarce.

Studies on the MI requirements of different aquatic animals have revealed the following results. Adding 800 mg·kg^−1^ MI to the high‐fat diet of hybrid grouper (♀*Epinephelus fuscoguttatus* × ♂*Epinephelus lanceolatus*) significantly increased the SGR of the fish [21]. When the MI level in the basal diet of *Micropterus salmoides* reached 375 mg·kg^−1^, the WGR, SGR, and feed efficiency ratio (FER) significantly improved [19]. Supplementing the basal diet with 1.44 g·kg^−1^ MI increased the WGR of *Litopenaeus vannamei* [22]. The FBW, WGR, SGR, and CF of *Trachinotus ovatus* fed basal diets supplemented with 300–1200 mg·kg^−1^ MI were greater than those of the nonsupplemented group [20]. Feeding juvenile *Eriocheir sinensis* diets containing more than 1650 mg·kg^−1^ MI significantly increased their FBW, WG, and SGR [10]. For *Cyprinus carpio* var. Jian, MI supplementation at 535.8 mg·kg^−1^ in the basal diet significantly improved the percentage weight gain (PWG). Cubic polynomial regression analysis revealed that the optimal MI level required for weight gain in juvenile *Cyprinus carpio* var. *Jian* was 518.0 mg·kg^−1^ [15]. In the present study, supplementation with 125 or 250 mg·kg^−1^ MI in the HCHF diet increased the FBW, WGR, and SGR of Yellow River carp, while decreasing the FCR. Adding 250 or 500 mg·kg^−1^ MI to the HCHF diet reduced the HSI of the fish. Cubic polynomial regression analysis indicated that the optimal MI supplementation levels in the HCHF diet for achieving optimal growth performance and minimum FCR in Yellow River carp were 251 and 260 mg·kg^−1^, respectively. These results demonstrate that the dietary MI requirements of different fish and shrimp species vary, but MI supplementation can promote the growth of aquatic animals.

### 4.2. Regulatory Effects of MI on Glucose Metabolism in Yellow River Carp

In animals, MI derivatives can act as intracellular second messengers to mediate cellular signal transduction processes. Variations in the inositol content within an organism accordingly alter the signal regulatory processes mediated by it.

The PI3K‐protein kinase B (PI3K‐Akt) pathway is the core pathway for insulin signal transduction in animals and exerts downstream regulatory effects on the insulin regulatory network [23]. After feeding, the blood glucose concentration of the organism increases, and insulin binds to the α‐subunit of the insulin receptor (IR) on the cell membrane, inducing a conformational change in the IR. This further triggers the cross‐phosphorylation and activation of tyrosine residues in the intracellular β‐subunit. The activated IR specifically binds to the phosphotyrosine‐binding (PTB) domain of IR substrate (IRS) in the cytoplasm, recruiting IRS to the vicinity of the cell membrane [24]. IRS subsequently binds to and activates the PI3K/Akt signaling pathway, ultimately promoting the translocation of glucose transporters to the cell membrane and inhibiting key gluconeogenic enzymes, thereby maintaining the homeostasis of glucose metabolism in the organism [25]. Glycogen synthase kinase 3β (GSK3β) and Forkhead Box O1 (FoxO1) are core molecular targets downstream of the PI3K‐Akt signaling pathway. As key negative regulators of insulin signal transduction, they inhibit insulin‐mediated metabolic effects through specific molecular mechanisms, jointly maintaining cellular glycolipid metabolic homeostasis [26]. Activated Akt can inactivate GSK3β via phosphorylation, thereby relieving the inhibitory effect on GS and promoting glycogen synthesis [27].

The present study revealed that appropriate MI supplementation in the HCHF diet significantly reduced the blood glucose concentration of Yellow River carp, which is consistent with the reported hypoglycemic effect of MI [28]. MI inhibited hepatic glycogen synthesis and promoted glycogenolysis, thereby reducing the excessive hepatic glycogen deposition induced by the HCHF diet. Western blot analysis of hepatopancreas tissues revealed that after MI supplementation, the protein expression levels of p‐PI3K, p‐PI3K/PI3K, p‐Akt, and p‐Akt/Akt were significantly increased. These results suggest that MI supplementation in the HCHF diet may help maintain glucose metabolic homeostasis in Yellow River carp, possibly through processes involving elevated PI3K‐Akt phosphorylation and modified glycogen metabolism.

In addition, Akt phosphorylates the transcription factor FOXO1 to prevent its nuclear localization in the liver, thereby suppressing the expression of gluconeogenesis‐related genes, such as PEPCK and G6Pase, and inhibiting gluconeogenesis, thus reducing the blood glucose level of the organism [29]. In the present study, supplementation of 250 mg·kg^−1^ MI in the HCHF diet significantly reduced the PEPCK activity. In contrast, supplementation with either 250 or 500 mg·kg^−1^ MI led to peak activities of the key glycolytic enzymes PFK and PK in the hepatopancreas of Yellow River carp. These results indicate that an appropriate MI dosage can significantly increase the glycolytic capacity of Yellow River carp. Specifically, PFK catalyzes the conversion of fructose‐6‐phosphate to fructose‐1,6‐bisphosphate, whereas PK catalyzes the formation of pyruvate from phosphoenolpyruvate. As the core rate‐limiting enzymes in the glycolytic pathway, the simultaneous upregulation of their activities can synergistically accelerate the catabolic process of glucose. Besides, our study revealed that MI treatment significantly up‐regulated *hk* mRNA expression, whereas no significant alteration was observed in HK enzyme activity. There is no simple linear correspondence between gene transcript levels and functional enzyme activity. Multiple factors, including post‐transcriptional regulation, post‐translational modification, and sampling time‐window effects, may lead to such decoupling. The exact molecular mechanism remains to be further explored in future investigations.

### 4.3. Regulatory Effects of MI on Lipid Metabolism in Yellow River Carp

The widespread application of HCHF diets in aquaculture has become a major contributor to nutritional fatty liver disease in fish, thereby inducing metabolic disorders and ectopic fat deposition. To maintain lipid metabolic homeostasis, fish have evolved multiple regulatory pathways, among which bile acid‐mediated regulation of lipid digestion and metabolism represents one of the core pathways. After fish ingest lipids, bile acids stored in the gallbladder are secreted into the intestine to facilitate lipid emulsification and subsequent absorption processes [30]. The level of bile acid metabolism is affected by the dietary composition. For example, studies in Yellow River carp have shown that HCHF feeding reshapes the composition of the bile acid pool, elevates secondary bile acid levels, and disturbs the homeostasis of bile acid metabolism in the hepatopancreas and intestine [12]. Bile acid metabolism is governed mainly by the negative feedback regulation of the FXR/SHP signaling pathway, and this regulatory process occurs primarily in the liver. The mechanism underlying the negative feedback regulation of bile acid synthesis by the FXR/SHP pathway has been well elucidated in mammals [31]. When the expression of FXR/SHP is inhibited, its transcriptional repression of cholesterol 7α‐hydroxylase (CYP7A1) and sterol 12α‐hydroxylase (CYP8B1) is relieved, thereby activating the activities of these two enzymes and further promoting the synthesis of primary bile acids. Conversely, elevated bile acid levels resulting from enhanced synthesis specifically activate hepatic FXR/SHP expression, which then inhibits the transcription of CYP7A1 and CYP8B1 in the liver through a negative feedback mechanism. This ultimately reduces the synthesis of bile acids in the hepatopancreas and maintains metabolic homeostasis [32]. It is worth noting that the regulatory characteristics of the FXR/SHP pathway on bile acid metabolism differ between fish and mammals. Existing studies on teleosts have only reported partial correlative phenotypes, and the complete negative feedback regulatory mechanism has not been fully elucidated. Available evidence indicates that hepatic FXR and SHP participate in the maintenance of bile acid homeostasis in fish. Studies on zebrafish have confirmed that key components of hepatic bile salt synthesis and ileal transport pathways are conserved and under FXR control; the expression of *cyp7a1*, the rate‐limiting gene for primary bile acid synthesis, is upregulated in *fxr* knockout mutants (*fxr^−^/^−^*) [30]. Dietary bile acid intervention trials conducted in largemouth bass, Yellow River carp, and other fish species have observed that hepatic FXR activation is accompanied by upregulation of *shp* and decreased transcript levels of *cyp7a1* [12, 33]. Despite these correlative observations, direct experimental evidence for the transcriptional repression of *cyp7a1* and *cyp8b1* by FXR/SHP remains lacking in fish at this stage.

In the present study, MI was found to participate in the regulation of lipid metabolism in fish by modulating the bile acid metabolism. At both the gene and protein levels, MI significantly downregulated the expression levels of FXR and SHP in the hepatopancreas of Yellow River carp while increasing the expression of CYP7A1 and CYP8B1. Therefore, MI may alleviate the lipid metabolic disorders induced by HCHF diets, possibly by enhancing bile acid synthesis and optimizing lipid metabolism.

Studies on mammals have shown that inositol deficiency slows the synthesis rate of phosphatidylinositol, whereas activation of the phosphatidylinositol synthesis pathway can reduce fat accumulation in intestinal tissues [34]. Other studies have shown that dietary MI supplementation significantly inhibits the increase in liver weight induced by high‐glucose diets in rats; alleviates the increasing trend in total hepatic lipid, TG, and cholesterol levels; and decreases serum TG levels simultaneously [8]. In a high‐fructose diet‐induced fatty liver model in rats, MI exerted beneficial effects on reducing lipid accumulation. It further suppressed the expression of downstream genes related to fructose catabolism and fat synthesis by downregulating the expression levels of sterol regulatory element‐binding protein 1c (SREBP1c) and carbohydrate‐responsive element‐binding protein (ChREBP), ultimately effectively alleviating hepatic enlargement and reducing TG accumulation in the livers of rats [35].

Studies on aquatic animals have also confirmed that MI ameliorates fat accumulation in fish. Waagbø and Lie [36] reported that the plasma TG content of Atlantic salmon (*Salmo salar*) was negatively correlated with dietary MI levels. Regression analysis revealed that, on the basis of growth performance indices, supplementing the diet of parrot fish (*Oplegnathus fasciatus*) with exogenous MI at approximately 100 mg·kg^−1^ significantly improved growth performance, reduced the feed conversion ratio, decreased hepatic lipid deposition, and protected liver function [37]. H&E staining in the present study showed that the HCHF diet induced hepatocyte hypertrophy and cytoplasmic vacuolation in the hepatopancreas of Yellow River carp. Dietary MI supplementation ameliorated these hepatocyte morphological abnormalities and reduced the cell area ratio. These morphological observations suggest that MI could alleviate HCHF‐induced hepatocyte morphological alterations. Meanwhile, the present results showed that appropriate MI supplementation in HCHF diets significantly reduced the contents of TG and T‐CHO and elevated the level of HDL‐C in the serum and hepatopancreas of common carp. Furthermore, MI inhibited the expression of fatty acid synthesis‐related genes (*fas* and *acc-1*) and fatty acid transport‐related genes (*cd36* and *fatp*), thereby alleviating hepatic lipogenesis. These results are consistent with previous studies in mammals and aquatic animals, further verifying the conserved regulatory effect of MI on animal lipid metabolism.

It is worth noting that the fatty acid synthesis pathway does not merely mediate lipid storage. Lipids generated by this pathway also serve as essential substances for cellular structure construction, cell signaling, and the maintenance of normal physiological functions. Accordingly, excessive inhibition of fatty acid synthesis may disrupt metabolic homeostasis. In the present study, under HCHF feeding conditions, MI intervention suppressed the enhancement of fatty acid synthesis induced by the HCHF diet. Future studies could further explore the effects of MI supplementation under normal dietary conditions to investigate its potential impacts on the fatty acid synthesis pathway, so as to comprehensively understand the regulatory effects of MI on lipid metabolism.

### 4.4. Optimal MI Supplementation Level in the Present Study

When fish suffer from MI deficiency, a series of physiological abnormalities and growth disorders often occur. For example, one study revealed that the optimal MI supplementation level in the diet of common carp (*Cyprinus carpio*) is 770.5 mg·kg^−1^ [11]. A dosage below this threshold significantly reduces the activities of lysozyme and acid phosphatase, as well as the contents of complement C3 and C4 in the head kidney and spleen. The same study also revealed that the endogenous MI synthesis of common carp cannot meet the demands of its own growth, and exogenous supplementation through the diet is therefore essential [11]. However, some fish species can synthesize sufficient MI endogenously to meet their physiological requirements without the need for additional supplementation in the diet. For example, sunshine bass (*Morone chrysops* ♀ × *Morone saxatilis* ♂) [38], channel catfish (*Ictalurus punctatus*) [39], and Pacific abalone (*Haliotis discus hannai Ino*) [6] are capable of endogenously producing adequate MI to support their growth and tissue physiological functions, thus requiring no dietary MI supplementation.

It is evident that significant interspecific and intraspecific differences exist in the endogenous MI synthesis capacity of fish. Previous studies have confirmed that among aquatic animals, carnivorous and omnivorous fish present significantly higher MI requirements than herbivorous fish do [40, 41]. The core reason lies in the fact that compound feeds for carnivorous and omnivorous fish usually contain a relatively high proportion of lipids. These fish need to ingest more MI to provide necessary regulatory support for fat catabolic pathways, thereby efficiently utilizing lipid substances and maintaining the bodily lipid metabolic homeostasis. In contrast, feeds for herbivorous fishes are dominated by plant‐based ingredients with low lipid contents, leading to a correspondingly reduced demand for MI in mediating fat metabolism. The regulatory factors underlying such differences are relatively complex. In addition to their feeding habits, they are closely associated with the growth stage, physiological status, genetic background, aquaculture environmental conditions, and dietary nutrient composition of fish [11]. Given the diversity of these influencing factors, further systematic research is still needed to clarify the precise MI requirements of aquatic animals.

Regarding the evaluation indicators for dietary MI requirements, a unified standard has not yet been established. Most studies adopt growth performance and hepatic MI accumulation as the core evaluation criteria, as these parameters can directly reflect the supportive effect of MI on animal growth and the accumulation balance status of the organism. In addition, some studies also use serum physiological and biochemical indices and key tissue enzyme activities as criteria for evaluating MI requirements. For the HCHF diet of Yellow River carp, different MI supplementation gradients were used in the present study. After data on growth performance, serum biochemical indices, and key enzyme activities related to glycolipid metabolism in the hepatopancreas that exhibited significant differences among the groups were obtained, cubic polynomial regression analysis was further employed to determine the optimal response relationships between key indicators and the MI dosage. This study ultimately recommends an optimal MI supplementation range of 230–475 mg·kg^−1^ for Yellow River carp fed this type of HCHF diet.

## 5. Conclusion

This study demonstrated that MI supplementation in a HCHF diet could significantly improve the growth performance of Yellow River carp, reduce the FCR and HSI, effectively regulate glycolipid metabolism indices in the serum and hepatopancreas, and alleviate the hepatocyte morphological abnormalities induced by the HCHF diet. This regulatory effect is associated with the PI3K/Akt signaling pathway and the FXR/SHP bile acid metabolic pathway, along with the altered expression of key enzymes and genes related to glycolipid metabolism. Collectively, these changes alleviate metabolic imbalance and hepatopancreatic impairment. On the basis of a comprehensive analysis of growth performance, physiological and biochemical indices, and enzyme activities, the optimal supplementation level of MI in the HCHF diet is recommended to range from 230–475 mg·kg^−1^. This study provides theoretical support for elucidating the metabolic regulatory mechanism of MI in fish and offers a reference for the prevention and control of glycolipid metabolic disorders and the development of high‐efficiency and eco‐friendly compound feeds in freshwater aquaculture.

## Author Contributions

**Xiao Yan**: project administration, conceptualization, writing – review and editing, funding acquisition. **Xin Long**: investigation, formal analysis, visualization, writing – original draft. **Rumeng Qiao** and **Shaoyang Zhi**: software. **Peng Pang**: formal analysis. **Junli Wang:** methodology, funding acquisition. **Jiali Mi**: supervision. **Mingming Niu**: conceptualization. **Chaobin Qin** and **Liping Yang**: methodology. **Guoxing Nie**: project administration, supervision, funding acquisition.

## Funding

This study was supported by the Natural Science Foundation of Henan (Grant 242300421114), the National Natural Science Foundation of China (Grants 32373142 and 32373149), and the Special Fund for Henan Agriculture Research System (Grant HARS‐22‐16‐S).

## Conflicts of Interest

The authors declare no conflicts of interest.

## Supporting Information

Additional supporting information can be found online in the Supporting Information section.

## Supporting information


**Supporting Information** Table S1: The primers of quantitative real‐time PCR.

## Data Availability

The data will be made available upon request.
