# Supplementary material for: Dietary Myo‐Inositol Improves Growth Performance and Glycolipid Metabolism in Yellow River Carp (Cyprinus carpio haematopterus) Fed High‐Carbohydrate‐High‐Fat Diets: Involvement of PI3K/Akt and FXR/SHP Pathways
Source: Aquac Nutr. 2026 Oct 25;2026:4324808. doi: 10.1155/anu/4324808 (PMC13695874; doi:10.1155/anu/4324808)
Supplement: Supplementary file 1 [file ANU-2026-4324808-s001.docx]

| **Gene** | **GeneBank ID** | **Sequence (5′-3′)(sense)** | **Sequences(3′-5′)(antisense)** |
| --- | --- | --- | --- |
| *18s* | LN591197.1 | F: GAGACTCCGGCTTGCTAAAT | R: CAGACCTGTTATTGCTCCATCT |
| *fxr* | XM_042729372.1 | F: CCCCTACAGCCATCAGT | R: GACCGACAATGCTCCCT |
| *shp* | XM_042741839.1 | F: GAAGTTCTGGAGTCTGGATT | R: GGATGAAGAGGCACAAGG |
| *cyp7a1* | XM_042718130.1 | F: CTACCTGACCCTCTTTGG | R: CGTGGAGCATTGTCTTGG |
| *cyp7b1* | XM_019073965.2 | F: CTTGCCATTGCTTGTCAT | R: GTATTCGGCTATCGTTCC |
| *hnf4α* | XM_019071428.2 | F: CAGGATTAGCACCAGAAGAT | R: TGTCGCCATTCAAGATAGG |
| *foxo1* | XM_042739020.1 | F: ACGGCTCACCTTATCTCA | R: CTTGCTGTTGTTGTCCATAG |
| *tgf5* | XM_042764258.1 | F: GCATTACGAACGCTACCTGTG | R: TGGAAGAGTTACGAGATGTGGT |
| *fgf19* | XM_042728520.1 | F: GTGGGAAAACTGTATGGATCGC | R: AAGGGCTCCATGTTTGCTTG |
| *hk* | XM_019097065.2 | F: CGTTGTAGCCGTAGTGAATGA | R: GCTACCCGTTCCTGCAATTA |
| *pk* | XM_042744551.1 | F: GATATGGGCTCTGCCTTCAT | R: TCCGATGGTGCAGATGATTC |
| *pfk* | XM_042766957.1 | F: CGTTCGACAGGAATTTTGGC | R: TTCATGCCGATCACACAAGC |
| *fbpase* | AF427864.1 | F: CCTGCCATCGGTGAGTTTAT | R: CCATCCTCTGGGAATTTCTTCT |
| *pepck* | KP250869.1 | F: ACTCTTTGGGCAGACCTTTAC | R: CTGTCTGGTGTCAGGAAGATG |
| *g6pase* | AF427863.1 | F: GAGGCCTTCAACAGACAGAAA | R: GAGCTTTGAGAAGCAGGTACAA |
| *gys-1* | XM_019090903.1 | F: TTTACTACACATGCCACCCTAC | R: TCTCTCCTGCCTCTTGATCTAT |
| *pygl* | XM_042737361.1 | F: TGGTTGACGACGATGCTTTC | R: ACTGCGCAAACTTCAGCTTG |
| *glut1* | XM_042726469.1 | F: CCCTCGATTTCTCCTCATCATC | R: GTCTGCTCTCCTCCTTCATTTC |
| *gcga* | XM_019065527.2 | F: TTCTGAGGGAACTTTCTCCAAC | R: GTTCATGAGCCACTGAACAAAG |
| *gcgb* | XM_042764332.1 | F: GGCACATTCTCAAACGATTACAG | R: GTTCATTAACCACTGAACGAAGTC |
| *ins* | XM_019099888.2 | F: GTGGTCCAACAGGTTTCTTCTA | R: AAGTCAGCCACCTCAGTTTC |
| *ir* | XM_042776936.1 | F: GATCTGCATCCCTCGTTTCA | R: CCATGTTCTCCAGGTCCATATC |
| *irs-1* | XM_042739891.1 | F: GCATCACTCTTCCCACCATTA | R: CATCCCTGGTCAGTCCAATATC |
| *pi3k* | XM_019085737.2 | F: GAAGATGACGACTGGAGAG | R: GCCTGTAGTGACTGATGAG |
| *akt* | XM_042774924.1 | F: ACTGTTATTGAGCGCACCTT | R: TCCATTGGCTCCTCCTCTTC |
| *gsk3β* | XM_042774357.1 | F: ATCAGGTGCATGAGGAGTTGAG | R: AGCAGAGCATTTCCAAACGC |
| *ampkα1* | XM_019104472.2 | F: TGCGAGAAGTTTGAGTGCAC | R: TTCATAATGCGCCGGTTGTC |
| *ampkα2* | XM_042777568.1 | F: AAATGTGTGCCAGCCTCATC | R: ATGTTTGTGGCGCTACTGTG |
| *fas* | GQ466045.1 | F: GACAGGCCGCTATTGCTATT | R: TGCCGTAAGCTGAGGAAATC |
| *acc-1* | XM_042757417.1 | F: GTCACTGGCGTATGAGGATATT | R: TCCACCTGTATGGTTCTTTGG |
| *srebp-1* | XM_042730308.1 | F: CGTCTGCTTCACTTCACTACTC | R: GGACCAGTCTTCATCCACAAA |
| *pparγ* | FJ849064.1 | F: AAGTCACCGAATTCGCCAAG | R: TGCCGTCTTTGTTCATGAGG |
| *hsl* | XM_042776500.1 | F: GCAGGTGGTAACCTGTGTATAA | R: TTAGCAGAGTTGCTGGGTATG |
| *cpt1* | JQ361077.1 | F: CAGATGGAAAGTGTTGCTAATGAC | R: TGTGTAGAAGTTGCTGTTGACCA |
| *lpl* | FJ716101.1 | F: CGCTCCATTCACCTGTTCAT | R: GCTGAGACACATGCCCTTATT |
| *aco* | XM_042743917.1 | F: GGAGATACCTGTCGTGGATTTC | R: GGCCTTTCTTAGTTCGTCACT |
| *cd36* | XM_019107844.2 | F: GAGACAGACACTCAGCAGATTG | R: TGGATGACGCCGTGAACTT |
| *fatp* | XM_019081506.1 | F: AATCAGCCGAAGAAGGACAC | R: AATCCACAGGCACCCGTCTT |

**Table S1** | The primers of quantitative real-time PCR
